# Biotechnological challenges: The scope of genome editing

**DOI:** 10.5935/1518-0557.20200038

**Published:** 2021

**Authors:** Natacha Salomé Lima, A Gustavo Martínez

**Affiliations:** 1 Consejo Nacional de Investigaciones Científicas y Técnicas (CONICET). Universidad de Buenos Aires. Facultad de Psicología, Buenos Aires, Argentina; 2 Medicina Reproductiva Fertilis, Laboratorio de Biología de la Reproducción, Boulogne, Buenos Aires, Argentina

**Keywords:** biotechnology, genome, CRISPR, edition

## Abstract

CRISPR/Cas9 can be considered as the biotechnological breakthrough of the century. Genome editing technologies have developed in a vertiginous way. While the genome editing of species, including animals, plants and bacteria has become a commonly used method, the application of CRISPR-Cas9 in human embryos has led to debates and interdisciplinary discussions. This brings multiple challenges for both scientists and those who must regulate the use of these techniques.

## INTRODUCTION

In the origins of the Renaissance period, Thomas More looked forward to a future where humanity would reach a fair existence. For centuries, even during the first half of the XX century, the Utopian ideas advocated for an emancipated humanity. After the Second World War, Auschwitz and the death fields, Hiroshima and Nagasaki, the Utopian ideas of Kant's Perpetual Peace were dismissed.

At the end of the Second World War, after the Nuremberg trials (1945-1946), the Code of Medical Ethics was published, containing a series of principles that regulate clinical research involving human beings (consent, social benefit, previous outcomes that justify the carrying out of the experiment, among others.) The purpose of the existing Code and future ones was to establish an appropriate relationship between benefits and risks, between autonomy and informed consent, between access to the benefits of scientific research and protection of new generations.

Most international documents have a declaratory force that is not fully reflected in actions, having a more formal than practical value. The conclusions are frequently non-binding, that is to say, they appear to be recommendations or pieces of advice.

One paradigmatic case to analyze ethics in relation to the scientific practice is Henrietta Lacks's case in the United States. In February 1951, she was diagnosed with cervical cancer at Johns Hopkins hospital. Once the tumor was discovered, it seemed unlike anything doctors had seen before. Prior to the treatment, Dr. Howard Jones removed cells from the carcinoma for research purposes, without the patient's consent. The sample gave origin to the first immortal cell line, known as HeLa cells. Henrietta died in October 1951 at the age of 31. Almost 20 years later, in the early 1970s, her family began to be unexpectedly visited by researchers who wanted to take blood samples to analyze and study the genetic family history. Her family was surprised, as nobody knew anything about Henrietta's cultivated cells. Nobody else in the family had the features that made Henrietta's cells unique.

These cells were the first ones grown in a laboratory, and what made them so special was that they did not die after a few cell divisions; they were called ‘immortal’. HeLa cells began to be used for different purposes: in 1954, John Salk used them to develop the poliomyelitis vaccine. Since HeLa cells began to be produced on a large scale, they have been used for research in cancer, AIDS, the effects of radiation and toxic substances, gene therapies and countless other scientific pursuits, representing a big stride for biomedical and biological research with more than 11,000 patents.

This story poses not only legal, but most importantly, ethical questions regarding the biological tissue rights and the scope of benefits for biomedical research; furthermore, it constitutes a background of what would be one of the most ambitious, eliciting, and known as the ‘genomics era’: the publication of the Human Genome Project (Collins *et al*., 1998).

The new biotechnological developments grew rapidly since the discovery of the DNA by James Watson and Francis Crick in 1953, up until 2000 when Bill Clinton -president of the United States at that time- and scientist Craig Venter announced that the human gene had been mapped. Indeed, there was much expectation of improving the human physiology through direct intervention of the genetic chain.

Ancient questions such as “what life is” or “what a human being is” should be read in light of the new paradigms and its conditions of emergence, with special focus on the sociocultural contexts, where the use of technologies is advocated as a means of approximation to the living entities from a biological point of view, together with the possibility of improving human beings that has pervaded along the history of humanity (Digilio, 2016).

## CRISPR-Cas: BACKGROUND

CRISPR/Cas9 can be considered as the biotechnological breakthrough of the century. Genome editing technologies have developed in a vertiginous way. While the genome editing of species, including animals, plants and bacteria has become a commonly used method, the application of CRISPR-Cas9 in human embryos has led to debates and interdisciplinary discussions.

There are several genome-editing techniques. One of them is “CRISPR-Cas9 system” (Hsu *et al*., 2014; Cox *et al*., 2015) based in programmable nucleases known as Cas nucleases - a CRISPR associated protein (Scharenberg *et al*., 2013; Stoddard, 2011; Urnov *et al*., 2010) and in the clustered regularly interspaced short palindromic repeats associated with these nucleases (CRISPR sequences).

In 1987, for the first time in history, a set of 29 nucleotide repeats that were interspaced by 32 nucleotides, within a coding DNA fragment present in *Escherichia coli,* was registered (Ishino *et al*., 1987). They were called CRISPR sequences (clustered regularly interspaced short palindromic repeats). Later research demonstrated that this structure, after being transcribed as RNA and associated to a Cas nuclease, known CRISPR-Cas system, constitutes a bacterial and archaeal adaptive mechanism of immunity and resistance against entities carrying invasive genetic material that enter the body (Ishino *et al*., 1987; Mojica *et al*., 2000; Jansen *et al*., 2002).

Since 2011, the CRISPR-Cas system applications began to be tested. In 2013, several groups of scientists, using this mechanism, successfully modified the genome in mammalian cells by the setting up of repair mechanisms (Cong & Zhang, 2015; Mali *et al*., 2013). From that moment onwards, each of the components of the system had been thoroughly studied and analyzed (Jinek *et al*., 2014; Nishimasu *et al*., 2014), eventually achieving a more refined genome editing technique.

The CRISPR-Cas9 system has been used to modify the genomes of a broad variety of species, including cultured human cells (Cong & Zhang, 2015), bacteria (Jiang *et al*., 2013a), amphibians (Nakayama *et al*., 2013), rodents (Wang *et al*., 2013), plants (Jiang *et al*., 2013b) and others.

The CRISPR-Cas9 technique, developed by researchers Emmanuelle Charpentier and Jennifer Doudna when studying molecular mechanisms of defense in Bacteria and Archaea, has been used in laboratories around the world for its advantages in comparison with other genome editing techniques.

In August 2012, Science magazine published the article “A Programmable Dual- RNA-Guided DNA Endonuclease in Adaptive Bacterial Immunity” (Jinek *et al*., 2014), describing how the bacterial system known as CRISPR-Cas9 could be used to manipulate genes in living organisms in a relatively simple and cheaper way than the methods used up to that moment. The authors described the activity of Cas9 protein that can find, cleave and degrade certain sections of deoxyribonucleic acid (DNA) of the cell, and proposed to take advantage of that function as a genetic engineering technology together with CRISPR, a system firstly used in *Escherichia coli* in 1987 by a group of Japanese scientists (Ishino *et al*., 1987).

Jennifer Doudna defined CRISPR-Cas9 as a useful system for scientists to eliminate or introduce specific DNA sequences in cells with great precision. The tool has two components: a Cas9 enzyme and a guide RNA (gRNA), made up of 20 nucleotides that are used to target a specific point in the double strand, to make a cleavage, which is codified from DNA flanked sites in CRISPR sequences.

The research conducted by Charpentier and Doudna is considered a breakthrough in Biotechnology, as it opened the possibility to correct defective genes; thus, eradicating diseases. It was considered Science's 2015 Breakthrough of the Year and the first out of ten scientific events of Nature; they also won Princess of Asturias Award for Technical and Scientific Research. However, the experiment and its application with therapeutic aims in human beings have been questioned.

The debate got intensified in April 2015, when Protein & Cell magazine released a research done by a group of scientists at Sun Yat-sen University in Guangzhou, China previously rejected by Nature and Science, which consisted in the use of CRISPR-Cas9 to alter the genome of human tripronuclear embryos (not viable) and to modify the gene responsible for Beta thalassemia, a blood disorder (Liang *et al*., 2015). Researchers explain their work with genome editing of human tripronuclear zygotes non-viable with the purpose of demonstrating, for the first time, the technique used in human embryos and its effectiveness in the correction of the gene responsible for β-thalassemia (Liang *et al*., 2015). Huang argues that, non-viable human embryos used in the study constitute the closest model to normal human embryos and state that they decided to show their results to the world so that people knew what really happened with this model, instead of arguing without evidence (Cyranoski & Reardon, 2015). Lanphier *et al*. (2015), when referring to Huang's publication, contend that we need to stop this type of research and we should discuss the path we are taking.

## TWO SIDES OF THE SAME COIN: HE'S GENETIC EXPERIMENT

At the end of 2018, after the Chinese researcher He Jiankui released a video on YouTube announcing the birth of two twin girls by using CRISPR-Cas9 technology, the world became shocked. The project was aimed at disabling the CCR5 gene in the embryo genome with the hope of making the subject immune to HIV, smallpox and cholera (MIT Report). He Jiankui explained that he modified the embryos of seven couples, of whom, only one managed to have a successful pregnancy, eventually giving birth to twin baby girls. A few days later, the news sparked a global reaction. Some scientists condemned He's research -including Feng Zhang, one of the inventors of genome-editing technique- and called for a global moratorium until the relevant safety conditions were granted to continue with the editing of human embryos.

Many aspects of this case are worth reflecting upon: the researchers chose to issue the outcomes in the social media, the procedures for selecting the subjects who participated in the experiment were unclear, and none of the research participants had the possibility of agreeing on unpredictable risks. It is no coincidence that the development of this phenomenon took place in China, which apart from being a leading country in genetic development (Raposo, 2019); its inhabitants have resented the birth restriction and the one-child policy for several years. The experiment of He and his team does not conform to any legal basis that, despite being not thoroughly described (Nie, 2018; Nie & Cheung, 2019), determined the prohibition of research in human embryos obtained by in vitro fertilization after day 14 and its implantation in the uterus (Raposo, 2019).

## BIOETHICAL CONSIDERATIONS SINCE CRISPR-Cas9 APPLICATION IN VIABLE EMBRYOS

One of the first issues is determining what the scope of CRISPR-Cas transformations can be and according to which purposes (Digilio, 2016).

A second issue is differentiating therapeutic uses of technologies (the use of technologies for genome editing aimed at treatment or cures a disease) for enhancement purposes. Nowadays, the editing of adult somatic cells by the introduction of a healthy gene in a tissue is already authorized for therapeutic aims. Uterus-interventions during fetal development or, *in ovo* (zygote), pose ethical questions because these modifications can produce a hereditary change and such change can be passed on to future generations.

He's genetic experiment can be analyzed as a paradigmatic case in light of three categories: 1) enhancement, 2) medical benefits and 3) the unsatisfied medical need for standard treatments.

When genome editing techniques are used not for treating an embryo disease but for enhancing the embryo with a genetic or phenotypic trait that is not present, that is to say, an additional trait, such as being immune to VIH virus, this technique can be considered as an enhancement. If we think that the enhancement is ethically acceptable as a means to restore ‘normalcy’, then He's genetic experiment fell into a misuse as for conducting it, healthy embryos were selected with the aim of transferring a trait (eliminating the CCR5 gene) that was not prevalent in the community. Besides, even if the procedure were successful, only genetic disorders caused by one gene can be corrected; however, the majority of existing disorders are polygenic.

The proposal enhancement is a possibility to restore ‘normalcy’, by eliminating a gene related to a disease presents at least two issues:

1) Off-target effects: the modifications that can be triggered after the elimination of that gene, most of which remain unknown. Due to its unpredictable character and the impossibility of evaluating exactly the risks, it cannot be thought of as a medical benefit. The context of He's experiment disregard the threshold between risks and benefits. Genome editing would be the unique option to conceive a healthy child by modifying the mutations that cause severe diseases and that cannot be prevented by any other way, for example in the case of chorea-Huntington disease.
2) Value the influence of the environmental component, knowing that there are no human characteristics determined solely by the genome; it always implies an interaction between the genome and the environment. Not acknowledging the environmental component can lead to promoting a genetic determinism that, associated with the logic of market behavior, attempts to set the DNA as the main source of health and wellbeing of the community.

## NORMATIVE CHALLENGES: PRECAUTIONARY PRINCIPLE AND MORATORIUM SPIRIT

The phenomenon under analysis has sparked more legal questions than answers. The International Declarations Soft Law by nature, because of their non-binding character, must be interpreted in light of each context of application. In this interplay, it is decisive to analyze the scope of the *Precautionary principle* and with it, its ethical place within scientific research.

Emerging from environmental considerations, the principle of precaution has evolved to become an ethical principle of greater scope. In its elemental form, the precautionary principle constitutes a strategy to deal with scientific uncertainties in risk evaluation and management (UNESCO- United Nations Educational, Scientific and Cultural Organization & World Commission on the Ethics of Scientific Knowledge and Technology, 2005).

Risk evaluation is a convergence point of interest, because it is there where science and public policies converge, in this sense, it is important to notice that risk evaluation is not a purely objective, it is a scientific, and not an apolitical process. The precautionary principle has questioned the convergence between science and legislation. Therefore, while scientists can provide data and analyses of risk sources (actual and perceived), they cannot determine the acceptable risk level or which measures can be justifiable in the attempt of preventing or diminishing the risk. This is the legislators' duty, mainly in close association with the stakeholders, including the industry and the public. While scientists can provide the information they have, regulation is a matter of politics.

When risks can be measured with a certain degree of confidence, and where the causal mechanisms between the substances or technology and the environmental or health effects have been relatively well studied, the legislator acts on the area of prevention.

The establishment of technical-scientific norms that might cause an impact on the community should be developed with the notion of distributive justice -as the common basis for strides in public policies, social justice and bioethics- to avoid these improvements being a new source of segregation between those who can have access to these techniques and those who cannot.

For some members of the European Group on Ethics in Science and New Technologies (EGE), genetic modification in the human germline with reproductive purposes cannot be ethically justifiable; therefore, it demands the application of Article 3 of the Charter of Fundamental Rights of the European Union, among others, due to the diffusing line between basic research and applied research. Other members of the EGE demand a moratorium of basic research that includes the genetic modification of human germline unless and until the legal framework adapts itself to the new possibilities (Cornejo Plaza, 2015).

In the international sphere, the Council of Europe (1997) in the Oviedo Convention, established the framework of European legislation and existing law, defined explicitly on Article 13, the Interventions on the human genome, that any human genome intervention may only be undertaken for preventive, diagnostic or therapeutic purposes and only if the ultimate aim is not to introduce modifications in the genome of any descendants.

In the three UNESCO Declarations -whose judicial nature is soft law- Universal Declaration on the Human Genome and Human Rights (UNESCO, 1998); International Declaration on Human Genetic Data (UNESCO, 2004); Universal Declaration on Bioethics and Human Rights (UNESCO, 2006) claim for the protection of human dignity, fundamental liberties and human rights.

While the ethical perspective links the applications of somatic cells with the adequate risk management for physical integrity or health, avoiding being disproportionate concerning the pathology aimed at curing and pretending to obtain the greatest certainty that any undesirable or unforeseen consequences will occur, does not affect other DNA sites (off-target), the answer provided by the law field -besides the principles of respect for human dignity and fundamental liberties- is also grounded on the precautionary principle. Facing the possibility of health risk, a risk assessment and a risk management should be carried out. While the former is a technical and scientific instance, the latter is an eminently political and judicial issue.

## FUTURE CONSIDERATIONS

It is inevitable that these new technologies be applied in the short term to medicine due to, as always, the fact that scientific development advances faster than its regulation. Therefore, the future poses the necessity of developing a working system that enables us to apply the new technologies in an ethical way, considering that the pace of development is greater than the pace in which these technologies can be regulated.
